# Rare forms of cerebral amyloid angiopathy: pathogenesis, biological and clinical features of CAA-ri and iCAA

**DOI:** 10.3389/fnins.2023.1219025

**Published:** 2023-07-10

**Authors:** Benedetta Storti, Maria Magdalena Gabriel, Stefan Sennfält, Isabella Canavero, Nicola Rifino, Laura Gatti, Anna Bersano

**Affiliations:** ^1^Cerebrovascular Unit, Fondazione IRCCS Istituto Neurologico Carlo Besta, Milan, Italy; ^2^Department of Neurology, Hannover Medical School, Hannover, Germany; ^3^Department of Neurology, Karolinska University Hospital, Stockholm, Sweden

**Keywords:** cerebral amyloid angiopathy (CAA), iatrogenic cerebral amyloid angiopathy (iCAA), brain hemorrhage, CAA-related inflammation (CAA-ri), beta amyloid

## Abstract

Thanks to a more widespread knowledge of the disease, and improved diagnostic techniques, the clinical spectrum of cerebral amyloid angiopathy (CAA) is now broad. Sporadic CAA, hereditary CAA, CAA-related inflammation (CAA-ri) and iatrogenic CAA (iCAA) create a clinical and radiological continuum which is intriguing and only partially discovered. Despite being relatively rare, CAA-ri, an aggressive subtype of CAA with vascular inflammation, has gained growing attention also because of the therapeutic efficacy of anti-inflammatory and immunomodulating drugs. More recently, diagnostic criteria have been proposed for an unusual variant of CAA, probably related to an iatrogenic origin (iCAA), toward which there is mounting scientific interest. These atypical forms of CAA are still poorly known, and their recognition can be challenging and deserve to be pursued in specialized referral centres. The aim of this brief review is to focus current developments in the field of rare forms of CAA, its pathogenesis as well as clinical and biological features in order to increase awareness of these rare forms.

## Introduction

Cerebral amyloid angiopathy (CAA) is characterized by deposition of amyloid-β (Aβ) in cortical and leptomeningeal vessels. The clinical features are lobar intracerebral hemorrhage (ICHs), progressive cognitive impairment, transient neurological episodes (TFNEs) (Viswanathan and Greenberg, 2011; Charidimou et al., 2012), and epilepsy (Tabaee Damavandi et al., 2023). Imaging findings of CAA include multiple strictly lobar cerebral microbleeds (CMBs) (Pasi et al., 2018), cortical superficial siderosis (cSS) or subarachnoid hemorrhage (SAH), white matter hyperintensities (WMHs), enlarged perivascular spaces in the centrum semiovale (CSO-PVS), and cortical atrophy (Samarasekera et al., 2012; Sharma et al., 2018). A definite diagnosis requires a neuropathologic confirmation (Greenberg and Vonsattel, 1997), but probable and possible CAA can be diagnosed on clinical and neuroimaging grounds (Greenberg and Charidimou, 2018), with relatively high sensitivity, e.g., by using MRI sequences sensitive to paramagnetic material (T2* sequences: GRE, echo-planar, SWI) such as hemoglobin deposits (Knudsen et al., 2001; Linn et al., 2010). As CAA is increasingly recognized worldwide, the definition of the clinical, radiological and biological phenotype is evolving, and diagnostic criteria are continuously updated. Within the spectrum of CAA, some subgroups of patients with diverse clinical presentations and characteristic radiological findings are emerging. CAA patients presenting with subacute cognitive impairment, seizures or headache, rather than intracerebral or subarachnoid hemorrhage, and with brain MRI and neuropathological evidence of inflammation, have been reported (Greenberg et al., 2010; Chung et al., 2011); this peculiar phenotype is known as CAA-related inflammation (CAA-ri) (Yamada, 2015; Auriel et al., 2016; Singh et al., 2022). The possibility of treatment with immunomodulatory and anti-inflammatory drugs makes its prompt identification imperative (Kozberg et al., 2021). Interestingly, in the last decade, several cases of iatrogenic Creuztfeld Jakob and concomitant CAA have been observed in patients exposed to cadaveric material many years earlier. This had occurred by administration of human pituitary-derived growth hormone (Jaunmuktane et al., 2015; Ritchie et al., 2017; Cali et al., 2018) or human-derived dura during major neurosurgical procedures (Frontzek et al., 2016; Hamaguchi et al., 2016; Cali et al., 2018). Thereafter, cases of patients with similar exposure, but without CJD, were reported (Hervé et al., 2018; Jaunmuktane et al., 2018) leading to a hypothesis of iatrogenic CAA (iCAA) (Banerjee et al., 2019; Greenberg and Charidimou, 2023). Proposed diagnostic criteria for this form, which seems to more often affect younger individuals than the sporadic form, have recently been published (Jaunmuktane et al., 2018) and a relatively large collection of Dutch cases has been described (Kaushik et al., 2023). This review article aims at providing an overview of the current state of knowledge of new amyloid angiopathy forms, increasing clinician awareness of the broad CAA spectrum.

## CAA-related inflammation: a severe, but potentially treatable form of CAA

CAA-ri is an aggressive condition with specific clinical and neuroradiological features, characterized by an inflammatory reaction to cerebral vascular deposits of Aβ (Salvarani et al., 2016). Table 1 summarizes the key elements of the disease. Several recent reviews have compiled hundreds of reported cases, revealing a wide range of clinical features and findings on imaging and other diagnostic tests (Castro Caldas et al., 2015; Corovic et al., 2018; Theodorou et al., 2023). The mean age at diagnosis is around 70 and the sex distribution is roughly equal (Castro Caldas et al., 2015; Corovic et al., 2018; Theodorou et al., 2023). CAA-ri most commonly presents with chronic to subacute cognitive impairment (in 50–70%), headache (in approximately 30%), seizures (in approximately 35%), and often various focal neurological symptoms; about 10–20% present acutely (Castro Caldas et al., 2015; Corovic et al., 2018; Theodorou et al., 2023). Cerebral MRI demonstrates microbleeds (CMBs) (in 40–96%) at T2* or SWI sequences and diffuse WMHs (in 60–98%) at T2/fluid-attenuated inversion recovery (FLAIR) sequences, often with contrast enhancement, and with an even distribution between the frontal, temporal, parietal, and occipital lobes (Castro Caldas et al., 2015; Corovic et al., 2018; Theodorou et al., 2023). Cerebrospinal fluid (CSF) examination shows elevated protein in most cases (65–80%), while there is an elevated white cell count of >5 cells/mm3 in 45–65% of cases (Castro Caldas et al., 2015; Corovic et al., 2018). The diagnosis is challenging due to the varying clinical presentation and lack of specific non-invasive tests. While a definite diagnosis requires brain biopsy or autopsy, a set of criteria similar to those for CAA, including lobar CMBs, cSS, WMHs and clinical features, has recently been proposed to identify probable cases, one report demonstrating a sensitivity of 82% and a specificity of 97% (Auriel et al., 2016). CAA is often identified during diagnostic workup of CAA-ri but might also be known for years prior to the inflammatory exacerbation (Yang et al., 2023). Although there are similarities between CAA-ri and primary CNS vasculitis (PACNS) there are differences suggesting that these are distinct entities: CAA-ri patients tend to be older, more likely to present with cognitive impairment, seizures and intracerebral hemorrhage (Salvarani et al., 2013). However, the underlying mechanism behind CAA-ri has not yet been satisfactorily elucidated. Several mechanisms have been proposed including (1) the accumulation of Aβ because of inflammation or (2) the induction of inflammation by the Aβ aggregates (Bogner et al., 2014). Interestingly, anti-β-amyloid antibodies have been found in CSF (DiFrancesco et al., 2011; Piazza et al., 2013) and blood (Hermann et al., 2011) of CAA-ri patients which might suggest an immunological basis. Also, imaging features like those found in CAA-ri are sometimes found in patients treated with therapeutic anti-β-amyloid antibodies: amyloid-related imaging abnormalities (ARIA) which has mostly been observed in the context of recent treatment of Alzheimer disease (Jeong et al., 2022). One study reported their presence in 17% of treated subjects of which a fourth experienced symptoms (e.g., headache and confusion) (Sperling et al., 2012). However, the role of anti- β-amyloid antibodies in the pathogenesis of CAA-ri is unclear and no systematic testing has been performed to date. Also, a possible clue to the pathophysiology is the strong association between CAA-ri and the ApoE ε4/ε4 genotype, which was present in 76.9% (10/13) of CAA-ri patients versus 5.1% (2/39) in those with non-inflammatory CAA (Kinnecom et al., 2007). The ApoE ε4/ε4 genotype is also seen more frequently in patients with ARIA (Sperling et al., 2012). There is little systematic evidence evaluating treatment strategies for CAA-ri, but the effectiveness of immunosuppressive therapy has been observed in relatively large patient series (Castro Caldas et al., 2015; Corovic et al., 2018; Regenhardt et al., 2020). The mainstay of treatment is corticosteroids, administered in 80% of patients and cyclophosphamide which is used in 30% of cases (Castro Caldas et al., 2015; Corovic et al., 2018). After a mean follow up of 2 years 50% of patients, irrespective of treatment strategy, are alive with no or mild disability while 35% are dead. It is unclear if there are differences in outcome between patients treated with corticosteroids alone or combinations of other agents.

**Table 1:** Key elements of rare forms of cerebral amyloid angiopathy compared to CAA.

	CAA-ri	i-CAA	CAA
Pathogenesis	Not fully understood. Current hypothesis: (a) Aβ deposition because of inflammation; (b) inflammatory reaction to cerebral vascular deposits of Aβ; (c) coexistence of Aβ and vascular inflammation	Prion like transmission of Aβ by human pituitary derived growth hormone, prior neurosurgical intervention with exposure to cadaveric material	Sporadic or hereditary deposition of Aβ in cortical and leptomeningeal vessels
Age at diagnosis	Mean age at diagnosis ~66 years	Symptom onset before the age of 55 years is possible, depending on exposure and latency; however diagnosis should be considered in people aged 55 years or above	Incidence is strongly age dependent; diagnosis is made in individuals over 55 years of age
Clinical presentation	Usually subacute cognitive impairment, focal neurological signs, seizures or headache, rather than ICH	TFNEs, focal seizures (with or without secondary generalization), cognitive impairment not attributable to another causes including AIS	ICH, cognitive impairment, TFNEs, epilepsy, headache
MRI findings in T2* sequences: GRE, echo-planar, SWI	CMBs, T2 FLAIR hyperintensities, often asymmetrical with contrast enhancement and confluent large distribution between the frontal, temporal, parietal, and occipital lobes	Multiple lobar CMBs, cSS (focal or disseminated), SAH, WMHs, CSO-PVS, cortical atrophy	Multiple lobar CMBs, cSS (focal or disseminated), SAH, WMHs, CSO-PVS, cortical atrophy
Diagnostic criteria	Similar to Boston 2.0 criteria, but definite diagnosis requires brain biopsy or autopsy	UCL diagnostic proposed criteria; definite diagnosis requires brain biopsy or autopsy	Boston 2.0 criteria; definite diagnosis requires brain biopsy or autopsy
CSF	Elevated protein, pleocytosis; rarely anti-β-amyloid antibodies	Reductions of Aβ − 42 and Aβ − 40.	Reduction up to 50% in levels of Aβ − 42 and Aβ − 40. Possible mildly increased total tau levels
ApoE genotype	Mostly ε4/ε4	ε3/ε3 predominance	Carriers of alleles ε2 and ε4 appear to have greater risk for CAA-related ICH
Therapy	Immunomodulatory and anti-inflammatory drugs, steroids	No therapy known, reduction of risk factors is proposed: i.e. hypertension, antithrombotic therapy, head injury	Reduction of risk factors: antithrombotic therapy, hypertension, potential head injury
Outcome	High effectiveness of immunosuppressive therapy	Data published to date suggest a better prognosis and evolution compared to sporadic CAA	Unfavorable features including lobar ICH, recurrent ICH and presence of cSS indicates much higher risk of ICH

FLAIR, Fluid attenuated inversion recovery; CAA, Cerebral amyloid angiopathy; CAA-ri, CAA-related inflammation; i-CAA, iatrogenic cerebral amyloid angiopathy; MRI, magnetic resonance imaging; GRE, gradient echo sequences; SWI, susceptibility weighted imaging; CSF, cerebrospinal fluid; ApoE, Apolipoprotein E; Aβ, amyloid-β; AIS, acute ischemic stroke; ICH, intracerebral haemorrhage; TFNE, transient neurological episode; CMBs, cerebral microbleeds; cSS, cortical superficial siderosis; SAH, subarachnoid haemorrhage; WMH, white matter hyperintensities; ARIA, amyloid-related imaging abnormalities; CSO-PVS, enlarged perivascular spaces in the centrum semiovale; PANCS, primary angiitis of the central nerval system.

## Iatrogenic cerebral amyloid angiopathy (iCAA): a new clinical concept

Iatrogenic cerebral amyloid angiopathy (iCAA) represents a rare but increasingly reported spectrum of amyloid pathology, characterized by an accumulation of Aβ in leptomeningeal and small cortical vessels, assumed to originate from a prion-like transmission after a previous neurosurgical intervention or cadaveric human growth factor intake (Banerjee et al., 2022). According to the proposed diagnostic criteria for iCAA, clinical and radiological features are consistent with a diagnosis of sporadic CAA; nonetheless, instrumental analyses suggestive of amyloid pathology (positivity at PET with amyloid tracer, decrease in abeta40 and abeta42 levels in CSF) may be helpful in supporting the diagnosis, especially when a peculiar young clinical onset before the age of 55 has been observed (Banerjee et al., 2022). Based on case series, diagnosis is most often made at a latency of three to four decades after exposure, affected patients being identified during single or repetitive intracranial hemorrhages, cognitive decline, seizures or transient neurological deficiency (Kaushik et al., 2023). Depending on the age at exposure and the latency of clinical and radiological features, patients can present at a relatively young age (<55) but might also be affected at an advanced stage of life. Considering the lack of long-term follow up data, a gradual progressive cognitive decline is rarely described, but the condition rather seems to predispose to the occurrence of clinical events such as intracranial hemorrhages (Kaushik et al., 2023). The main proposed underlying pathophysiological mechanism is seeding and aggregation in a prion-like manner (Jaunmuktane et al., 2015) where contamination with misfolded protein cascades furthers aggregation which consecutively leads to widespread structural and functional disturbance. This hypothesis is underlined by the diffuse distribution of Aβ irrespective of the location of the trigger intervention. Experiments in rodents have suggested that peripheral inoculation of human derived Aβ leads to deposit of amyloid in vessel walls (Eisele et al., 2010; Hamaguchi et al., 2021), whereas the transmission routes in humans are proposed to originate from cadaveric dura graft (Lyodura) (Frontzek et al., 2016; Hamaguchi et al., 2016; Kovacs et al., 2016; Cali et al., 2018; Kaushik et al., 2023), cadaveric human growth hormone intake (Jaunmuktane et al., 2015; Ritchie et al., 2017; Cali et al., 2018) or embolisation material (Banerjee et al., 2022) and contaminated medical devices (Gibbs et al., 1994; Collins et al., 1999; Bonda et al., 2016). Interventions do not only refer to brain or spinal cord related interventions, but also other surgical procedures with the need of cadaveric dura tissue are considered as potential transmission sources (Raposo et al., 2020; Caroppo et al., 2021). The notion of a prion-like mechanism is supported by eight described cases of individuals with iatrogenic Creutzfeldt-Jakob disease (iCJD) after treatment with cadaver derived human growth hormone (c-hGH) from pituitaries due to short stature (Jaunmuktane et al., 2015). Autopsy revealed Aβ disposition in the central nervous system parenchyma, in local brain regions or Aβ entrapment in prion protein plaques in these patients. Prion disease itself as the cause of precipitation or acceleration of Aβ pathology seems less likely since a control population of 116 individuals diagnosed with sporadic and variant CJD or inherited prion disease, did not demonstrate a comparable prevalence of intraparenchymal Aβ dispositions (Jaunmuktane et al., 2015). An alternative hypothesis involves an acquired failure of perivascular clearance of Aβ because of prior trauma or surgery. The breakdown of the complex cerebral fluid draining system might lead to amyloid deposition in capillary basement membranes of the cortex as proposed by fluorescent tracer studies (Hawkes et al., 2014). This concept might explain the prevalence of Aβ accumulation in young patients without a confirmed transmission route or neurosurgical intervention but with a history of traumatic brain injury (Nakayama et al., 2017). It is believed that genetic factors play a minor role in iCAA in young individuals; indeed, the exclusion of duplications or mutations in the *APP, PSEN1* and *PSEN2* genes has been suggested as diagnostic criteria (Banerjee et al., 2022). In many cases of iCAA an ApoE E3/E3 phenotype has been observed. To date, evidence for the attribution of iatrogenic Aβ pathologies to Alzheimer’s disease is discussed controversially. Although individual case reports indicate concomitant pathological levels of phosphorylated tau these are not present in the majority of the reported patients (Jaunmuktane et al., 2018), which is indispensable for confirming the diagnosis of Alzheimer’s disease (Jack et al., 2018). Nonetheless, the ability of Aβ to enhance tau pathology has been demonstrated *in vitro* and *in vivo* experiments before, and an impending confirmation of tau pathogens in the long run is possible (He et al., 2018; Vergara et al., 2019). Interestingly, in iCAA cases known so far, the onset of symptoms has been reported to occur at least 25 years after surgery. The age of exposure, on the other hand, is variable, suggesting a greater importance of latency, rather than early exposure. The use of Lyodura graft was discontinued before the 90s, when its relationship with the onset of Creutzfeldt-Jakob was attested. It is therefore reasonable that the first cases of iCAA were only described in recent years, some 30 years after its ban. It can be assumed that further cases of iCAA will be observed in the future, the manifestation of an unfortunate exposure that occurred up to four decades ago.

## Discussion

This review outlines recent developments and findings related to the amyloid pathology spectrum of diseases, highlighting the current lack of a comprehensive understanding. Many questions remain unanswered regarding the associated inflammatory condition known as CAA-ri. First of all, early recognition and treatment is crucial, therefore, a more precise definition of the phenotype and the identification of reliable biomarkers is necessary. Several diagnostic methods, including the detection of genotypes, CSF and blood biomarkers (including anti-Aβ autoantibodies), and various radiological techniques, could provide evidence for diagnosis and potentially eliminate the need for brain biopsy in suspected cases. Furthermore, greater knowledge of the therapeutic possibilities is necessary, as precise guidelines of CAA-ri treatment are still lacking and patient management can vary greatly from one hospital to another (Auriel et al., 2016). Clinical trials, preferably randomized with standardized enrolment and follow-up strategies with opportune outcome evaluation are required. Lastly, despite various hypotheses, the pathogenesis of CAA-ri is unexplained. While impaired Aβ clearance is thought to be the cause of CAA, CAA-ri may represent an immune response that targets Aβ aggregates. However, it is unknown whether there are factors making certain CAA patients susceptible to developing CAA-ri and the role of APOE e4 is largely undiscovered (Szidonya and Nickerson, 2023). Also, the long-term effects of immunosuppression regarding prognosis and adverse effects needs to be established. While available data on CAA-ri are scarce, only a relatively small number of clinical cases of iCAA have been reported so far. However, a failure of cerebral fluid drainage caused by amyloid overload can be hypothesized as a causative mechanism. Making the pathogenesis even more complex to understand, Aβ deposits could be seen in post-mortem analysis in variable locations. Despite appropriate suggestions for the underlying pathogenesis of the accumulation of Aβ in young individuals, it remains unclear why only few patients have been described worldwide so far, although the seeding transmission processes might have been triggered much more frequently. The lack of diagnostic methods and awareness might have played a key role. Therefore, to reduce misdiagnosis, standardization of investigative approaches has been recently proposed (Auriel et al., 2016; Banerjee et al., 2022). Missing clinical and imaging long term follow up data of iCAA patients limit our understanding of iCAA as a continuum with some forms being more prone to rapid progression of brain atrophy, hypometabolism and cognitive impairment. However, regular clinical follow-up of iCAA patients is needed to better understand the natural history of this condition and the similarities with sporadic CAA. Although the onset in young patients could support the diagnosis, iCAA (Rowe et al., 2013) might not be as obviously identifiable in people exposed at older ages, because sporadic disease could be a more likely diagnosis in these cases. In conclusion, international registries and large epidemiological studies are necessary to gather cases since CAA-ri and iCAA are rare entities within the CAA spectrum.

## Author contributions

BS, MMG, SS, IC, NR, LG, and AB conceived of the presented idea. BS, MMG, SS, and AB developed the manuscript. IC, LG, and NR supervised the findings of this work. All authors contributed to the article and approved the submitted version.

## References

[ref1] Auriel E. Charidimou A. Gurol M. E. Ni J. Van Etten E. S. Martinez-Ramirez S.. (2016). Validation of clinicoradiological criteria for the diagnosis of cerebral amyloid angiopathy-related inflammation. JAMA Neurol. 73, 197–202. doi: 10.1001/jamaneurol.2015.4078, PMID: 26720093

[ref2] Banerjee G. Adams M. E. Jaunmuktane Z. Alistair Lammie G. Turner B. Wani M.. (2019). Early onset cerebral amyloid angiopathy following childhood exposure to cadaveric dura. Ann. Neurol. 85, 284–290. doi: 10.1002/ana.25407, PMID: 30597599 PMC6492172

[ref3] Banerjee G. Samra K. Adams M. E. Jaunmuktane Z. Parry-Jones A. R. Grieve J.. (2022). Iatrogenic cerebral amyloid angiopathy: an emerging clinical phenomenon. J. Neurol. Neurosurg. Psychiatry 93, 693–700. doi: 10.1136/jnnp-2022-328792 35577510

[ref4] Bogner S. Bernreuther C. Matschke J. Barrera-Ocampo A. Sepulveda-Falla D. Leypoldt F.. (2014). Immune activation in amyloid-β-related angiitis correlates with decreased parenchymal amyloid-β plaque load. Neurodegener. Dis. 13, 38–44. doi: 10.1159/000352020, PMID: 24021982

[ref5] Bonda D. J. Manjila S. Mehndiratta P. Khan F. Miller B. R. Onwuzulike K.. (2016). Human prion diseases: surgical lessons learned from iatrogenic prion transmission. Neurosurg. Focus. 41:E10. doi: 10.3171/2016.5.FOCUS15126, PMID: 27364252 PMC5082740

[ref7] Cali I. Cohen M. L. Haik S. Parchi P. Giaccone G. Collins S. J.. (2018). Iatrogenic Creutzfeldt-Jakob disease with amyloid-β pathology: an international study. Acta Neuropathol. Commun. 6:5. doi: 10.1186/s40478-017-0503-z, PMID: 29310723 PMC5759292

[ref8] Caroppo P. Marucci G. Maccagnano E. Gobbo C. L. Bizzozero I. Tiraboschi P.. (2021). Cerebral amyloid angiopathy in a 51-year-old patient with embolization by dura mater extract and surgery for nasopharyngeal angiofibroma at age 17. Amyloid 28, 142–143. doi: 10.1080/13506129.2020.1854715, PMID: 33274650

[ref9] Castro Caldas A. Silva C. Albuquerque L. Pimentel J. Silva V. Ferro J. M. (2015). Cerebral amyloid angiopathy associated with inflammation: report of 3 cases and systematic review. J. Stroke Cerebrovasc. Dis. 24, 2039–2048. doi: 10.1016/j.jstrokecerebrovasdis.2015.04.015, PMID: 26163888

[ref10] Charidimou A. Gang Q. Werring D. J. (2012). Sporadic cerebral amyloid angiopathy revisited: recent insights into pathophysiology and clinical spectrum. J. Neurol. Neurosurg. Psychiatry 83, 124–137. doi: 10.1136/jnnp-2011-301308, PMID: 22056963

[ref11] Chung K. K. Anderson N. E. Hutchinson D. Synek B. Barber P. A. (2011). Cerebral amyloid angiopathy related inflammation: three case reports and a review. J. Neurol. Neurosurg. Psychiatry 82, 20–26. doi: 10.1136/jnnp.2009.204180, PMID: 20935328

[ref12] Collins S. Law M. G. Fletcher A. Boyd A. Kaldor J. Masters C. L. (1999). Surgical treatment and risk of sporadic Creutzfeldt-Jakob disease: a case-control study. Lancet 353, 693–697. doi: 10.1016/s0140-6736(98)08138-0, PMID: 10073510

[ref13] Corovic A. Kelly S. Markus H. S. (2018). Cerebral amyloid angiopathy associated with inflammation: a systematic review of clinical and imaging features and outcome. Int. J. Stroke 13, 257–267. doi: 10.1177/1747493017741569, PMID: 29134927

[ref14] DiFrancesco J. C. Brioschi M. Brighina L. Ruffmann C. Saracchi E. Costantino G.. (2011). Anti-Abeta autoantibodies in the CSF of a patient with CAA-related inflammation: a case report. Neurology 76, 842–844. doi: 10.1212/WNL.0b013e31820e773c, PMID: 21357837

[ref15] Eisele Y. S. Obermüller U. Heilbronner G. Baumann F. Kaeser S. A. Wolburg H.. (2010). Peripherally applied Abeta-containing inoculates induce cerebral beta-amyloidosis. Science 330, 980–982. doi: 10.1126/science.1194516 20966215 PMC3233904

[ref16] Frontzek K. Lutz M. I. Aguzzi A. Kovacs G. G. Budka H. (2016). Amyloid-β pathology and cerebral amyloid angiopathy are frequent in iatrogenic Creutzfeldt-Jakob disease after dural grafting. Swiss Med. Wkly. 146:w14287. doi: 10.4414/smw.2016.14287 26812492

[ref17] Gibbs C. J. Jr. Asher D. M. Kobrine A. Amyx H. L. Sulima M. P. Gajdusek D. C. (1994). Transmission of Creutzfeldt-Jakob disease to a chimpanzee by electrodes contaminated during neurosurgery. J. Neurol. Neurosurg. Psychiatry 57, 757–758. doi: 10.1136/jnnp.57.6.757, PMID: 8006664 PMC1072988

[ref18] Greenberg S. M. Charidimou A. (2018). Diagnosis of cerebral amyloid angiopathy: evolution of the Boston criteria. Stroke 49, 491–497. doi: 10.1161/STROKEAHA.117.016990, PMID: 29335334 PMC5892842

[ref19] Greenberg S. M. Charidimou A. (2023). Seed to bleed: iatrogenic cerebral amyloid angiopathy. Stroke 54, 1224–1226. doi: 10.1161/STROKEAHA.123.042583, PMID: 37035915 PMC10473030

[ref20] Greenberg S. M. Rapalino O. Frosch M. P. (2010). Case records of the Massachusetts General Hospital. Case 22-2010. An 87-year-old woman with dementia and a seizure. N. Engl. J. Med. 363, 373–381. doi: 10.1056/NEJMcpc1004364, PMID: 20660406

[ref21] Greenberg S. M. Vonsattel J. P. (1997). Diagnosis of cerebral amyloid angiopathy. Sensitivity and specificity of cortical biopsy. Stroke 28, 1418–1422. doi: 10.1161/01.str.28.7.1418 9227694

[ref22] Hamaguchi T. Kim J. H. Hasegawa A. Goto R. Sakai K. Ono K.. (2021). Exogenous Aβ seeds induce Aβ depositions in the blood vessels rather than the brain parenchyma, independently of Aβ strain specific information. Acta Neuropathol. Commun. 9, 1–17. doi: 10.1186/s40478-021-01252-0, PMID: 34507620 PMC8431898

[ref23] Hamaguchi T. Taniguchi Y. Sakai K. Kitamoto T. Takao M. Murayama S.. (2016). Significant association of cadaveric dura mater grafting with subpial Aβ deposition and meningeal amyloid angiopathy. Acta Neuropathol. 132, 313–315. doi: 10.1007/s00401-016-1588-3, PMID: 27314593

[ref24] Hawkes C. A. Jayakody N. Johnston D. A. Bechmann I. Carare R. O. (2014). Failure of perivascular drainage of β-amyloid in cerebral amyloid angiopathy. Brain Pathol. 24, 396–403. doi: 10.1111/bpa.12159, PMID: 24946077 PMC8029317

[ref25] He Z. Guo J. L. McBride J. D. Narasimhan S. Kim H. Changolkar L.. (2018). Amyloid-β plaques enhance Alzheimer's brain tau-seeded pathologies by facilitating neuritic plaque tau aggregation. Nat. Med. 24, 29–38. doi: 10.1038/nm.4443, PMID: 29200205 PMC5760353

[ref26] Hermann D. M. Keyvani K. van de Nes J. Weimar C. Wiltfang J. Nitsch R. M.. (2011). Brain-reactive beta-amyloid antibodies in primary CNS angiitis with cerebral amyloid angiopathy. Neurology 77, 503–505. doi: 10.1212/WNL.0b013e318227b250, PMID: 21775736

[ref27] Hervé D. Porché M. Cabrejo L. Guidoux C. Tournier-Lasserve E. Nicolas G.. (2018). Fatal Aβ cerebral amyloid angiopathy 4 decades after a dural graft at the age of 2 years. Acta Neuropathol. 135, 801–803. doi: 10.1007/s00401-018-1828-9, PMID: 29508058

[ref28] Jack C. R. Jr. Bennett D. A. Blennow K. Carrillo M. C. Dunn B. Haeberlein S. B.. (2018). NIA-AA research framework: toward a biological definition of Alzheimer's disease. Alzheimers Dement. 14, 535–562. doi: 10.1016/j.jalz.2018.02.018, PMID: 29653606 PMC5958625

[ref29] Jaunmuktane Z. Mead S. Ellis M. Wadsworth J. D. Nicoll A. J. Kenny J.. (2015). Evidence for human transmission of amyloid-β pathology and cerebral amyloid angiopathy. Nature 525, 247–250. doi: 10.1038/nature15369, PMID: 26354483

[ref30] Jaunmuktane Z. Quaegebeur A. Taipa R. Viana-Baptista M. Barbosa R. Koriath C.. (2018). Evidence of amyloid-β cerebral amyloid angiopathy transmission through neurosurgery. Acta Neuropathol. 135, 671–679. doi: 10.1007/s00401-018-1822-2, PMID: 29450646 PMC5904220

[ref31] Jeong S. Y. Suh C. H. Shim W. H. Lim J. S. Lee J. H. Kim S. J. (2022). Incidence of amyloid-related imaging abnormalities in patients with Alzheimer disease treated with anti-β-amyloid immunotherapy: a meta-analysis. Neurology 99, e2092–e2101. doi: 10.1212/WNL.0000000000201019.Erratum 36038268

[ref32] Kaushik K. van Etten E. S. Siegerink B. Kappelle L. J. Lemstra A. W. Schreuder F. H. B. M.. (2023). Iatrogenic cerebral amyloid angiopathy post neurosurgery: frequency, clinical profile, radiological features, and outcome. Stroke 54, 1214–1223. doi: 10.1161/STROKEAHA.122.041690, PMID: 37035916 PMC10121246

[ref33] Kinnecom C. Lev M. H. Wendell L. Smith E. E. Rosand J. Frosch M. P.. (2007). Course of cerebral amyloid angiopathy-related inflammation. Neurology 68, 1411–1416. doi: 10.1212/01.wnl.0000260066.98681.2e, PMID: 17452586

[ref34] Knudsen K. A. Rosand J. Karluk D. Greenberg S. M. (2001). Clinical diagnosis of cerebral amyloid angiopathy: validation of the Boston criteria. Neurology 56, 537–539. doi: 10.1212/wnl.56.4.537 11222803

[ref35] Kovacs G. G. Lutz M. I. Ricken G. Ströbel T. Höftberger R. Preusser M.. (2016). Dura mater is a potential source of Aβ seeds. Acta Neuropathol. 131, 911–923. doi: 10.1007/s00401-016-1565-x, PMID: 27016065 PMC4865536

[ref36] Kozberg M. G. Perosa V. Gurol M. E. van Veluw S. J. (2021). A practical approach to the management of cerebral amyloid angiopathy. Int. J. Stroke 16, 356–369. doi: 10.1177/1747493020974464, PMID: 33252026 PMC9097498

[ref37] Linn J. Halpin A. Demaerel P. Ruhland J. Giese A. D. Dichgans M.. (2010). Prevalence of superficial siderosis in patients with cerebral amyloid angiopathy. Neurology 74, 1346–1350. doi: 10.1212/WNL.0b013e3181dad605, PMID: 20421578 PMC2875936

[ref38] Nakayama Y. Mineharu Y. Arawaka Y. Nishida S. Tsuji H. Miyake H.. (2017). Cerebral amyloid angiopathy in a young man with a history of traumatic brain injury: a case report and review of the literature. Acta Neurochir. 159, 15–18. doi: 10.1007/s00701-016-3004-0, PMID: 27812816

[ref39] Pasi M. Marini S. Morotti A. Boulouis G. Xiong L. Charidimou A.. (2018). Cerebellar hematoma location: implications for the underlying microangiopathy. Stroke 49, 207–210. doi: 10.1161/STROKEAHA.117.019286, PMID: 29183952 PMC5742084

[ref40] Piazza F. Greenberg S. M. Savoiardo M. Gardinetti M. Chiapparini L. Raicher I.. (2013). Anti-amyloid beta autoantibodies in cerebral amyloid angiopathy-related inflammation: implications for amyloid-modifying therapies. Ann. Neurol. 73, 449–458. doi: 10.1002/ana.23857, PMID: 23625526

[ref41] Raposo N. Planton M. Siegfried A. Calviere L. Payoux P. Albucher J. F.. (2020). Amyloid-β transmission through cardiac surgery using cadaveric dura mater patch. J. Neurol. Neurosurg. Psychiatry 91, 440–441. doi: 10.1136/jnnp-2019-321927, PMID: 31959705

[ref42] Regenhardt R. W. Thon J. M. Das A. S. Thon O. R. Charidimou A. Viswanathan A.. (2020). Association between immunosuppressive treatment and outcomes of cerebral amyloid angiopathy-related inflammation. JAMA Neurol. 77, 1261–1269. doi: 10.1001/jamaneurol.2020.1782, PMID: 32568365 PMC7309570

[ref43] Ritchie D. L. Adlard P. Peden A. H. Lowrie S. Le Grice M. Burns K.. (2017). Amyloid-β accumulation in the CNS in human growth hormone recipients in the UK. Acta Neuropathol. 134, 221–240. doi: 10.1007/s00401-017-1703-0, PMID: 28349199 PMC5508038

[ref44] Rowe C. C. Bourgeat P. Ellis K. A. Brown B. Lim Y. Y. Mulligan R.. (2013). Predicting Alzheimer disease with β-amyloid imaging: results from the Australian imaging, biomarkers, and lifestyle study of ageing. Ann. Neurol. 74, 905–913. doi: 10.1002/ana.24040 24448836

[ref45] Salvarani C. Hunder G. G. Morris J. M. Brown R. D. Jr. Christianson T. Giannini C. (2013). Abeta-related angiitis: comparison with CAA without inflammation and primary CNS vasculitis. Neurology 81, 1596–1603. doi: 10.1212/WNL.0b013e3182a9f545, PMID: 24078731 PMC3806912

[ref46] Salvarani C. Morris J. M. Giannini C. Brown R. D. Jr. Christianson T. Hunder G. G. (2016). Imaging findings of cerebral amyloid angiopathy, abeta-related angiitis (ABRA), and cerebral amyloid angiopathy-related inflammation: a single-institution 25-year experience. Medicine (Baltimore) 95:e3613. doi: 10.1097/MD.0000000000003613, PMID: 27196463 PMC4902405

[ref47] Samarasekera N. Smith C. Al-Shahi S. R. (2012). The association between cerebral amyloid angiopathy and intracerebral haemorrhage: systematic review and meta-analysis. J. Neurol. Neurosurg. Psychiatry 83, 275–281. doi: 10.1136/jnnp-2011-300371, PMID: 22056966

[ref48] Sharma R. Dearaugo S. Infeld B. O'Sullivan R. Gerraty R. P. (2018). Cerebral amyloid angiopathy: review of clinico-radiological features and mimics. J. Med. Imaging Radiat. Oncol. 62, 451–463. doi: 10.1111/1754-9485.12726, PMID: 29604173

[ref49] Singh B. Lavezo J. Gavito-Higueroa J. Ahmed F. Narasimhan S. Brar S.. (2022). Updated outlook of cerebral amyloid angiopathy and inflammatory subtypes: pathophysiology, clinical manifestations, diagnosis and management. J. Alzheimers Dis. Rep. 6, 627–639. doi: 10.3233/ADR-220055, PMID: 36447738 PMC9661355

[ref50] Sperling R. Salloway S. Brooks D. J. Tampieri D. Barakos J. Fox N. C.. (2012). Amyloid-related imaging abnormalities in patients with Alzheimer's disease treated with bapineuzumab: a retrospective analysis. Lancet Neurol. 11, 241–249. doi: 10.1016/S1474-4422(12)70015-7, PMID: 22305802 PMC4063417

[ref51] Szidonya L. Nickerson J. P. (2023). Cerebral amyloid Angiopathy. Radiol. Clin. N. Am. 61, 551–562. doi: 10.1016/j.rcl.2023.01.009 36931769

[ref52] Tabaee Damavandi P. Storti B. Fabin N. Bianchi E. Ferrarese C. DiFrancesco J. C. (2023). Epilepsy in cerebral amyloid angiopathy: an observational retrospective study of a large population. Epilepsia 64, 500–510. doi: 10.1111/epi.17489, PMID: 36515439

[ref53] Theodorou A. Palaiodimou L. Malhotra K. Zompola C. Katsanos A. H. Shoamanesh A.. (2023). Clinical, neuroimaging, and genetic markers in cerebral amyloid angiopathy-related inflammation: a systematic review and meta-analysis. Stroke 54, 178–188. doi: 10.1161/STROKEAHA.122.040671, PMID: 36453271

[ref54] Vergara C. Houben S. Suain V. Yilmaz Z. De Decker R. Vanden Dries V.. (2019). Amyloid-β pathology enhances pathological fibrillary tau seeding induced by Alzheimer PHF in vivo. Acta Neuropathol. 137, 397–412. doi: 10.1007/s00401-018-1953-5, PMID: 30599077

[ref55] Viswanathan A. Greenberg S. M. (2011). Cerebral amyloid angiopathy in the elderly. Ann. Neurol. 70, 871–880. doi: 10.1002/ana.22516, PMID: 22190361 PMC4004372

[ref56] Yamada M. (2015). Cerebral amyloid angiopathy: emerging concepts. J. Stroke 17, 17–30. doi: 10.5853/jos.2015.17.1.17, PMID: 25692104 PMC4325636

[ref57] Yang J. Y. Chu Y. T. Tsai H. H. Jeng J. S. (2023). Amyloid and tau PET in cerebral amyloid angiopathy-related inflammation two case reports and literature review. Front. Neurol. 14:1153305. doi: 10.3389/fneur.2023.1153305, PMID: 37188315 PMC10175602

